# Quantitative Radiographic and Computed Tomography Assessment of Pediatric Aneurysmal Bone Cysts: Relative Transverse Expansion, Physeal Proximity, and Postoperative Outcomes

**DOI:** 10.3390/bioengineering13101110

**Published:** 2026-09-24

**Authors:** Sheng Jin, Kun Deng, Haoqi Cai, Yuchan Li, Zhigang Wang, Zhu Liu, Yuling Li

**Affiliations:** 1 Department of Orthopedic Surgery, Shanghai Children’s Medical Center, Shanghai Jiao Tong University School of Medicine, Shanghai 200127, China; 2 Department of Anesthesiology, Shanghai Children’s Medical Center, Shanghai Jiao Tong University School of Medicine, Shanghai 200127, China; 3 Department of Pediatric Urology, Xinhua Hospital, Shanghai Jiao Tong University School of Medicine, Shanghai 200092, China

**Keywords:** aneurysmal bone cyst, biomedical imaging, children, computed tomography, Hounsfield units, physis, quantitative imaging, relative transverse cyst index

## Abstract

Pediatric aneurysmal bone cysts (ABCs) are locally aggressive lesions with variable postoperative behavior, and quantitative imaging markers associated with healing or recurrence remain limited. This exploratory, retrospective, single-center study evaluated whether the relative transverse cyst index (RTCI), lesion-to-physis distance, and mean intralesional computed tomography (CT) attenuation were associated with radiographic outcomes after surgery. Twenty-four children with biopsy-confirmed primary ABCs treated between 2017 and 2023 underwent blinded dual-observer measurement of lesion dimensions, involved-bone length, physeal distance, and CT attenuation; outcomes were classified as complete healing, recurrence, or stable partial residual disease. RTCI differed across outcome groups (0.069 ± 0.024, 0.126 ± 0.046, and 0.114 ± 0.0534, respectively; *p* = 0.019, false-discovery-rate-adjusted *q* = 0.045), as did lesion-to-physis distance (4.58 ± 3.64, 1.04 ± 1.09, and 1.26 ± 1.95 cm; *p* = 0.023, *q* = 0.045). Mean CT attenuation did not differ among groups (*p* = 0.843). Greater relative transverse expansion and closer physeal proximity were associated with less favorable radiographic outcomes. These hypothesis-generating findings require validation in larger multicenter cohorts.

## 1. Introduction

Aneurysmal bone cyst (ABC) is a benign but locally aggressive bone neoplasm that occurs mainly in children and adolescents. Large pediatric series show that ABCs most often involve long bones and typically present during the first two decades of life [1]. Primary ABCs are characterized by blood-filled cystic spaces and, in many cases, USP6 rearrangements, whereas ABC-like changes may occur secondarily in other bone tumors [2,3,4]. Lesions may enlarge rapidly, thin or disrupt the cortex, cause pathological fracture, and extend toward a joint or open physis [2,3,5,6]. Pediatric series have reported recurrence rates of approximately one-quarter after surgery, with growth-plate complications in some patients [7]. Repeated treatment can affect bone stock, growth, function, and health-related quality of life, particularly for lesions near the pelvis or major joints [8].

Treatment is multidisciplinary and individualized according to lesion location, skeletal maturity, structural stability, pathological fracture, local vascularity, and the expected morbidity of treatment. Curettage with defect reconstruction remains common for accessible long-bone lesions, whereas sclerotherapy, selective arterial embolization, and other image-guided or minimally invasive techniques are used in selected patients [6,9,10,11,12,13,14,15,16,17,18,19,20]. Recent systematic reviews and large case series indicate that several approaches can achieve satisfactory local control, but recurrence varies with age, lesion location, treatment approach, imaging phenotype, outcome definition, and follow-up duration [5,9,10,11,12,13,14,15,16]. Thus, imaging serves not only diagnostic radiology but also orthopedic oncology, operative planning, interventional treatment, follow-up assessment, and quantitative phenotyping.

Imaging is central to the diagnosis, treatment planning, and follow-up of ABCs. Plain radiography, computed tomography (CT), and magnetic resonance imaging (MRI) are used to assess lesion expansion, cortical thinning or destruction, lobulation, internal septation, periosteal reaction, pathological fracture, fluid–fluid levels, enhancement, and extraosseous extension [2,3,5,21,22]. Outcome studies have also explored categorical or semiquantitative features such as Enneking stage, Capanna type, soft-tissue edema, fluid–fluid levels, and physeal involvement [5,23]. More broadly, methodological studies in biomedical imaging have shown that observer-dependent annotation and inter-device measurement concordance can materially affect quantitative imaging outputs, underscoring the need to establish reproducibility before interpreting imaging-derived biomarkers [24,25]. In children, absolute lesion size is often reported as a diameter or broad size category, although absolute size alone has not consistently been associated with recurrence [26,27]. This approach may be particularly limited because skeletal dimensions vary substantially with age and anatomical site, so the same lesion width may represent very different proportions of the host bone.

To address this scaling problem, we used the relative transverse cyst index (RTCI), defined as the maximum transverse lesion diameter divided by the involved-bone length. ABCs are characteristically expansile lesions with cortical thinning and enlargement of the host bone [2,3]; therefore, transverse expansion is a clinically recognizable part of their radiographic phenotype. RTCI does not represent lesion volume, but it provides a simple dimensionless estimate of transverse expansion relative to skeletal size. We also measured lesion-to-physis distance as a continuous variable because binary descriptors such as “physeal contact” may lose spatial information, and curettage close to an open growth plate may be constrained by the risk of physeal injury [23,26].

The internal composition of an ABC may provide a different type of quantitative information. Histologically, ABCs contain blood-filled spaces separated by fibrous septa with stromal cells, giant cells, hemosiderin, reactive bone, and variable mineralization [2,3,4]. Fluid–fluid levels arise when intracystic materials of different densities separate into layers and are commonly interpreted as evidence of hemorrhage and sedimentation. In the 2025 series of 258 ABCs, fluid–fluid levels were associated with a shorter time to recurrence, although not with a higher overall recurrence frequency [5]. This suggests that intracystic composition may carry information not captured by lesion dimensions alone.

CT attenuation provides a simple way to explore intracystic composition quantitatively. Hounsfield units (HUs) reflect X-ray attenuation and vary with material density and composition. Experimental and clinical CT studies of blood show that attenuation changes with hemoglobin and hematocrit, supporting a relationship between measured HU and the relative concentration of cellular blood components [28]. In an ABC, mean intralesional HU may therefore be influenced by the proportions of blood, serum, clot, proteinaceous fluid, cellular debris, septa, and mineralized tissue. It should not be interpreted as a direct biochemical measurement of cyst fluid; rather, it is an exploratory imaging surrogate for average intracystic density. To our knowledge, mean intralesional HU has not been systematically evaluated as an outcome-associated quantitative feature in pediatric primary ABC.

Accordingly, this study was designed as an exploratory, hypothesis-generating quantitative imaging analysis rather than as a clinical prediction study. We evaluated whether RTCI, lesion-to-physis distance, and mean intralesional CT attenuation were associated with postoperative radiographic outcomes in children with surgically treated primary ABCs. Age at surgery was retained as a clinically relevant secondary variable, and interobserver reliability was assessed to determine whether the imaging measurements could be reproduced consistently.

## 2. Materials and Methods

### 2.1. Study Design and Participants

This retrospective, single-center cohort study was conducted at Shanghai Children’s Medical Center. The study was approved by theIRB of Shanghai Children’s Medical Center Affiliated to Shanghai Jiao Tong University School of Medicine(SCMCIRB-K2026060-1; 16 March 2026), and informed consent was waived because only existing clinical records and imaging data were reviewed.

Children aged 18 years and younger who had undergone primary ABC surgery between January 2017 and December 2023 were screened. Inclusion criteria were (i) biopsy-confirmed primary ABC; (ii) preoperative radiographs and CT; and (iii) postoperative follow-up of at least 12 months. Exclusion criteria were secondary ABC-like changes associated with another bone neoplasm, spinal lesions, previous treatment at another institution, incomplete preoperative imaging, or inadequate follow-up. All eligible patients who met these criteria were included in the study. No a priori sample size was calculated.

Sex was abstracted from the medical record and summarized descriptively. Race or ethnicity was not used as a stratification variable in this single-center Chinese cohort. Pubertal maturity, Tanner stage, and menarchal status were not routinely documented in the retrospective records and therefore could not be analyzed. Because only five girls were included, sex- or maturity-stratified inferential analyses were not prespecified, as such subgroup estimates would be unstable.

### 2.2. Surgical Treatment and Follow-Up

All procedures were performed by pediatric orthopedic surgeons experienced in bone tumor surgery. After exposure of the lesion, the cyst was opened, its contents were evacuated, and the cavity was thoroughly curetted. A high-speed burr was used when necessary to extend intralesional clearance. The defect was reconstructed with a Wright artificial bone graft in all patients. Internal fixation was added when indicated by lesion location, pathological fracture, or mechanical instability: fifteen patients received intramedullary nails, five received PHP plates, and four underwent no internal fixation. Patients were assessed clinically and radiographically at 6 weeks, 3 months, 6 months, and 12 months, and annually thereafter. Complete healing was defined as radiographic consolidation with cortical remodeling and no progressive cystic component. Recurrence was defined as radiographic re-expansion of the lesion; recurrent cases underwent revision surgery with histological confirmation of ABC. Partial residual disease was defined as a persistent radiolucent component or incomplete cavity filling without progressive enlargement on serial follow-up imaging.

### 2.3. Quantitative Imaging Measurements

Preoperative measurements included maximum transverse lesion diameter, maximum longitudinal lesion diameter, involved-bone length, lesion-to-physis distance, and mean intralesional CT attenuation. Two observers performed all measurements independently and were blinded to each other. For radiographic measurements, the maximum transverse diameter used for RTCI was measured on the standard anteroposterior projection, perpendicular to the longitudinal axis of the involved bone; the involved-bone length was measured on the same projection. The mean of the two observer measurements was used for outcome comparisons. RTCI was calculated as the maximum transverse lesion diameter divided by the involved-bone length. This dimensionless ratio expresses transverse lesion expansion relative to skeletal size and anatomical site and was treated as a study-derived exploratory quantitative imaging metric rather than a validated ABC classification index.

Mean intralesional CT attenuation was measured on the axial CT image showing the largest cross-sectional area of the lesion. A region of interest (ROI) was manually drawn within the intramedullary cystic component to encompass as much of the lesion as possible while excluding surrounding cortical bone (Figure 1). The mean attenuation within the ROI was recorded in Hounsfield units (HUs). Lesion-to-physis distance was measured from the nearest lesion margin to the closest open physis on the CT plane that best demonstrated this relationship.

CT examinations were acquired as part of routine clinical care over the 2017–2023 study period rather than under a prospectively harmonized research protocol. Available acquisition information indicated a tube voltage of 100 kVp and a slice thickness of 0.625 mm for the analyzed studies, but scanner model information was not consistently retrievable. Other technical factors, such as tube current and reconstruction kernel, may therefore have varied across examinations. Because complete acquisition metadata were not available in the analytic dataset for protocol-level adjustment, this heterogeneity is particularly relevant to absolute HU measurements and was considered when interpreting CT attenuation results.

### 2.4. Statistical Analysis

Continuous variables are presented as mean ± standard deviation (SD), and categorical variables as number (percentage). Because of the small sample size and potential non-normality within the three outcome groups, four prespecified variables were compared with Kruskal–Wallis tests: RTCI, lesion-to-physis distance, mean CT attenuation, and age at surgery. Benjamini–Hochberg false-discovery-rate (FDR) correction was applied across these four overall comparisons. For variables that remained significant after FDR correction, pairwise Mann–Whitney U tests were performed with Holm adjustment. All tests were two-sided.

Interobserver reliability was assessed using the intraclass correlation coefficient (ICC), calculated using a two-way random-effects model for absolute agreement based on single measurements [ICC(2,1)], with bootstrap 95% confidence intervals [29]. Because the cohort included only eight recurrences, no multivariable prediction model, receiver operating characteristic-derived threshold, or machine learning classifier was developed. In response to the clinically intermediate partial-residual group, a post hoc sensitivity analysis combined recurrence and stable partial residual disease as an “unfavorable radiographic outcome” and compared this composite with complete healing using Mann–Whitney U tests for the same four variables; Benjamini–Hochberg FDR correction was again applied across the four sensitivity comparisons. Analyses were performed in Python 3.11 using SciPy, NumPy, and statsmodels.

## 3. Results

### 3.1. Patient Characteristics and Outcomes

Twenty-four children met the eligibility criteria. Mean age at surgery was 7.65 ± 2.65 years. Nineteen patients (79.2%) were male, and five (20.8%) were female. Sixteen patients (66.7%) presented with a pathological fracture. The proximal humerus was the most frequent site (9/24, 37.5%), followed by the proximal femur (5/24, 20.8%). Internal fixation was used in 20 patients (83.3%). Mean follow-up was 36.54 ± 6.88 months (range, 24–48 months). At the latest follow-up, 11 patients (45.8%) had complete radiographic healing, eight (33.3%) had developed recurrence, and five (20.8%) had stable partial residual disease without progressive enlargement (Table 1).

### 3.2. Relative Transverse Cyst Index

RTCI differed among the three outcome groups (Kruskal–Wallis *p* = 0.019; BH-FDR *q* = 0.045). Mean ± SD values were 0.069 ± 0.024 for complete healing, 0.126 ± 0.046 for recurrence, and 0.114 ± 0.054 for partial residual disease. The complete-healing and recurrence groups remained different after Holm correction (adjusted *p* = 0.008) (Table 2; Figure 2A). Pairwise comparisons involving partial residual disease were not statistically significant.

### 3.3. Lesion-to-Physis Distance

Lesion-to-physis distance also differed among outcome groups (Kruskal–Wallis *p* = 0.023; BH-FDR *q* = 0.045). Mean distance was 4.58 ± 3.64 cm in the complete-healing group, 1.04 ± 1.09 cm in the recurrence group, and 1.26 ± 1.95 cm in the partial-residual group. The difference between complete healing and recurrence remained significant after Holm adjustment (adjusted *p* = 0.044) (Table 2; Figure 2B).

### 3.4. CT Attenuation and Age

Mean intralesional CT attenuation did not differ among outcomes: 55.2 ± 12.9 HU in the complete-healing group, 51.4 ± 24.2 HU in the recurrence group, and 50.3 ± 8.3 HU in the partial-residual group (*p* = 0.843; *q* = 0.843). Mean age at surgery was 8.83 ± 2.63, 6.53 ± 2.05, and 6.83 ± 2.90 years, respectively; the overall age comparison was not statistically significant (*p* = 0.143; *q* = 0.191) (Table 2). The patient-level distributions of CT attenuation and age are shown in Figure 3.

### 3.5. Interobserver Reliability

Interobserver agreement was high for all quantitative measurements. ICC(2,1) was 0.979 for maximum transverse diameter, 0.999 for maximum longitudinal diameter, 0.978 for mean intralesional CT attenuation, >0.999 for bone length, and >0.999 for lesion-to-physis distance (Table 3; Figure 4). The high reliability of CT attenuation indicates that the absence of an outcome association was not explained by poor interobserver reproducibility.

### 3.6. Sensitivity Analysis Combining Recurrence and Partial Residual Disease

When recurrence and stable partial residual disease were combined as an unfavorable radiographic outcome (*n* = 13), the two principal imaging associations were preserved. Mean RTCI was 0.121 ± 0.048 in the unfavorable-outcome group versus 0.069 ± 0.024 in the complete-healing group (Mann–Whitney *p* = 0.0077; BH-FDR *q* = 0.015). Mean lesion-to-physis distance was 1.12 ± 1.41 cm versus 4.58 ± 3.64 cm, respectively (*p* = 0.0065; *q* = 0.015). Mean CT attenuation remained non-significant (*p* = 0.582; *q* = 0.582), and age at surgery showed only borderline evidence of a difference (*p* = 0.059; *q* = 0.079). Thus, combining the partial-residual group with recurrence did not reverse the direction or interpretation of the primary RTCI and physeal-distance findings (Table 4).

### 3.7. Representative Case Examples

Representative radiographic sequences illustrating the three predefined postoperative outcome categories are shown in Figure 5. Case 1 was a 6-year-old boy with a left proximal humeral aneurysmal bone cyst (ABC) who underwent curettage, bone grafting, and elastic intramedullary nail fixation; follow-up radiographs approximately 37 months after surgery demonstrated recurrent cystic expansion. Case 2 was an 11-year-old boy with a left humeral shaft ABC treated with curettage, bone grafting, and elastic intramedullary nail fixation; follow-up radiographs approximately 12.5 months after surgery demonstrated complete osseous consolidation and cortical remodeling. Case 3 was a 9-year-old boy with a right proximal humeral ABC treated with curettage and bone grafting without internal fixation; at approximately 24 months after surgery, a residual radiolucent component remained visible but showed no progressive enlargement and was therefore classified as stable partial residual disease.

## 4. Discussion

### 4.1. Principal Findings

Two quantitative imaging measurements differed across postoperative radiographic outcome groups in this cohort: RTCI and lesion-to-physis distance. Children who achieved complete healing had less transverse expansion relative to the length of the involved bone and a greater distance between the lesion and the nearest open physis. The partial-residual group had mean values closer to the recurrence group for both measurements, and the post hoc sensitivity analysis combining partial residual disease with recurrence preserved both associations. Mean CT attenuation was similar across outcomes despite high interobserver agreement. Representative case sequences showing these three outcome patterns are provided in Figure 5.

These findings describe associations rather than individual risk. The cohort was deliberately analyzed with a small number of prespecified variables rather than a multivariable prediction model. This approach reduces overfitting in a dataset with only eight recurrences, but the estimates remain imprecise and require external validation.

### 4.2. Relative Transverse Expansion

Expansion, cortical thinning, and enlargement of the host bone are major imaging features of ABC [2,3]. Prior outcome studies have investigated absolute lesion size, broad size categories, Enneking stage, Capanna type, physeal contact, and other categorized radiographic findings [5,23,26,27]. Recent pediatric bone-cyst cohorts have likewise emphasized that lesion location and structural imaging features can relate to postoperative outcome, although these studies differ in diagnosis mix, anatomical site, and treatment strategy [30]. Absolute lesion size has not been consistently correlated with recurrence in children, possibly because the same absolute lesion width can occupy a much larger proportion of a small child’s bone than an adolescent’s bone.

RTCI was developed to normalize transverse lesion expansion to the length of the involved bone. Mean RTCI was 0.126 in recurrent lesions and 0.069 in lesions that healed completely. A higher RTCI therefore indicates greater transverse expansion relative to skeletal size and may reflect more extensive local structural involvement, including cortical thinning and reduced residual bone stock. The ratio is simple, dimensionless, and obtainable from routine radiographs without segmentation software. However, it is not a volumetric measure and does not capture longitudinal extent, cortical eccentricity, or three-dimensional morphology. RTCI should therefore be interpreted as a normalized descriptor of lesion expansion rather than as a new classification system. Because the metric was derived in the present cohort, external validation is required to determine whether it provides information beyond absolute lesion dimensions across different anatomical sites.

### 4.3. Physeal Proximity

Shorter lesion-to-physis distance was also associated with a less favorable radiographic outcome. Previous studies have reported mixed findings regarding physeal proximity. Lin et al. found higher recurrence at juxtaphyseal sites in skeletally immature patients, whereas Başarır et al. and a recent pediatric series by Gettleman et al. did not identify a consistent association between physeal contact and recurrence [23,26,27]. Earlier pediatric series also documented physeal arrest as an important treatment-related complication [7]. These differences may partly reflect how the variable is defined: a binary contact/no-contact descriptor loses spatial information, whereas a continuous distance preserves the degree of separation from the growth plate.

The association is clinically plausible. Curettage near an open physis requires a balance between local clearance and preservation of growth potential. More conservative curettage on the physeal side may protect the growth plate but could leave residual lesion tissue. The present data cannot determine whether the observed association reflects this surgical constraint, local biology, or both, and they do not define a validated distance threshold.

### 4.4. CT Attenuation and Intracystic Composition

Mean intralesional CT attenuation was not associated with postoperative outcome. The reason for measuring HU was based on the hemorrhagic and heterogeneous architecture of ABC. Blood-filled spaces contain variable proportions of red cells, serum, clot, proteinaceous material, cellular debris, fibrous septa, hemosiderin, reactive bone, and mineralization [2,3,4]. Fluid–fluid levels are a visible manifestation of density separation within this mixture, and recent clinical data suggest that hemorrhagic imaging features may relate to lesion behavior: Karaca et al. reported a shorter time to recurrence in ABCs with fluid–fluid levels [5].

CT attenuation provides a continuous measure of average X-ray attenuation within the selected ROI. Blood attenuation is influenced by hemoglobin and hematocrit; a 2024 dual-energy CT study of more than 800 blood samples demonstrated positive correlations between CT attenuation and laboratory hemoglobin/hematocrit values [28]. In an ABC, mean HU may therefore partly reflect the balance between cellular blood products and lower-density fluid components. This interpretation is necessarily indirect. A single ROI averages a multiloculated lesion and cannot distinguish dependent blood products, supernatant fluid, septa, clot, or mineralized foci. The absence of an association between mean HU and outcome is therefore informative rather than a measurement failure: interobserver agreement for CT attenuation was high (ICC 0.978), making poor reproducibility an unlikely explanation. Mean attenuation may simply be too coarse to capture biologically relevant intracystic heterogeneity. Absolute HU values may also be influenced by acquisition parameters, reconstruction kernels, partial-volume effects, and ROI placement. Future studies could examine compartment-specific attenuation, attenuation histograms, or quantitative fluid–fluid-level characteristics; based on the present data, however, mean intralesional HU should not be considered a prognostic marker.

### 4.5. Reproducibility of the Imaging Measurements

All five quantitative imaging measurements showed high interobserver agreement. Reproducibility is essential before manually derived imaging measurements can be interpreted biologically or compared across outcome groups. The components of RTCI—maximum transverse lesion diameter and bone length—had ICCs of 0.979 and >0.999, respectively. Lesion-to-physis distance showed near-perfect agreement (>0.999), and CT attenuation remained highly reproducible (ICC 0.978) despite its lack of association with outcome. These findings underscore that measurement reliability and prognostic relevance are distinct properties and should be evaluated separately.

### 4.6. Clinical Context and Outcome Classification

All patients in this cohort were treated with curettage and bone grafting, with internal fixation added when mechanical stability required it. The study was not designed to compare surgery with sclerotherapy, selective arterial embolization, or other treatment strategies. Systematic reviews and comparative studies indicate that several approaches can achieve local control, and treatment selection should account for lesion site, skeletal maturity, mechanical stability, pathological fracture, and expected morbidity [6,9,10,11,12,13,14,15,16,17,18,19,27]. Long-term pediatric series, particularly for pelvic lesions, also emphasize functional outcome and quality of life in addition to radiographic recurrence [8,31]. A recent pediatric humeral ABC cohort further illustrates the importance of site-specific treatment and radiographic follow-up after curettage and reconstruction [32]. Partial residual disease was therefore retained as a distinct radiographic outcome rather than automatically being classified as recurrence. Its mean RTCI and lesion-to-physis distance were closer to those of the recurrence group than to those of the complete-healing group; because only five patients had this outcome, we added a sensitivity analysis combining partial residual disease with recurrence. The principal RTCI and physeal-distance associations were preserved. Longer follow-up remains necessary to determine whether stable residual disease represents durable remodeling or a precursor to later progression.

### 4.7. Strengths and Limitations

The main strength of this study is its focus on a small set of quantitative measurements obtainable from routine radiographs and CT without specialized segmentation software. Measurements were performed independently by two observers; all 24 patients had paired measurements, and interobserver reliability was formally quantified. The analysis was restricted to four prespecified primary comparisons with FDR correction rather than fitting a prediction model to only eight recurrences. Several limitations should be considered. First, the cohort was small, retrospective, single-center, and anatomically heterogeneous; fixation was selected according to mechanical need rather than assigned uniformly. Second, RTCI was derived in this cohort and has not been externally validated. Third, CT acquisition was not prospectively standardized across the seven-year study period, which may affect absolute HU values. Fourth, three-dimensional lesion volume was not analyzed. Recent pediatric ABC work has used lesion volume as a quantitative imaging variable [33], and volume could complement or confound a one-dimensional normalized index such as RTCI, particularly in irregular multiloculated lesions. Fifth, only five girls were included, preventing meaningful sex-stratified inference; pubertal maturity and menarchal status were not routinely documented, and race/ethnicity was not analyzed as a stratification variable in this single-center cohort. Finally, follow-up ranged from 24 to 48 months, so later progression of stable partial residual disease remains possible.

### 4.8. Future Directions

Future studies should prioritize prospective multicenter cohorts with harmonized radiographic and CT acquisition, predefined imaging planes, and complete scanner metadata. Three-dimensional segmentation could test whether lesion volume, surface geometry, cortical involvement, or volumetric distance to the physis adds information beyond RTCI. For CT attenuation, compartment-specific measurements, attenuation histograms, and quantitative characterization of fluid–fluid levels may capture intracystic heterogeneity more effectively than a single mean ROI. Longitudinal follow-up of partial residual disease is particularly important to determine whether it represents stable remodeling or delayed recurrence. Only after external validation in substantially larger cohorts should imaging thresholds or multivariable risk tools be considered for individual treatment planning.

## 5. Conclusions

In this exploratory cohort of 24 children with surgically treated primary ABC, greater relative transverse expansion and closer physeal proximity were associated with less favorable postoperative radiographic outcomes. Mean RTCI was 0.126 ± 0.046 in recurrent lesions versus 0.069 ± 0.024 in lesions that healed completely (overall *p* = 0.019; FDR-adjusted *q* = 0.045), while mean lesion-to-physis distance was 1.04 ± 1.09 versus 4.58 ± 3.64 cm (*p* = 0.023; *q* = 0.045). These associations were preserved when recurrence and partial residual disease were combined in a sensitivity analysis. Mean intralesional CT attenuation was highly reproducible but was not associated with outcome. RTCI and lesion-to-physis distance are simple quantitative imaging measures, but the present findings remain hypothesis-generating and require multicenter validation before either measure is used as a treatment threshold or individual risk score.

## Figures and Tables

**Figure 1:**
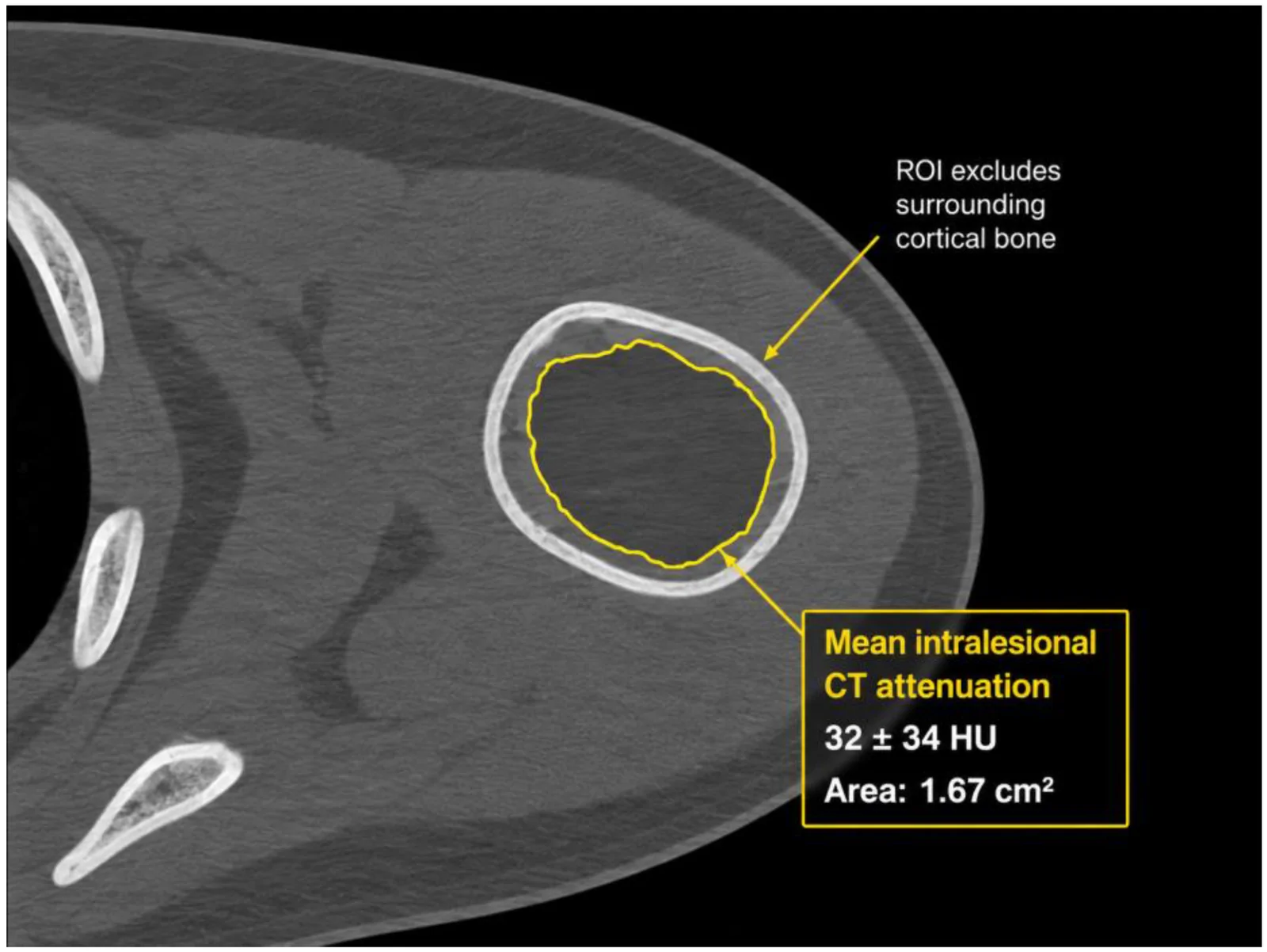
Representative method for measuring mean intralesional CT attenuation. The ROI is drawn within the intramedullary cystic component on the axial CT image showing the largest lesion cross-section. Surrounding cortical bone is excluded. The displayed HU value is illustrative of the measurement obtained from the selected ROI.

**Figure 2:**
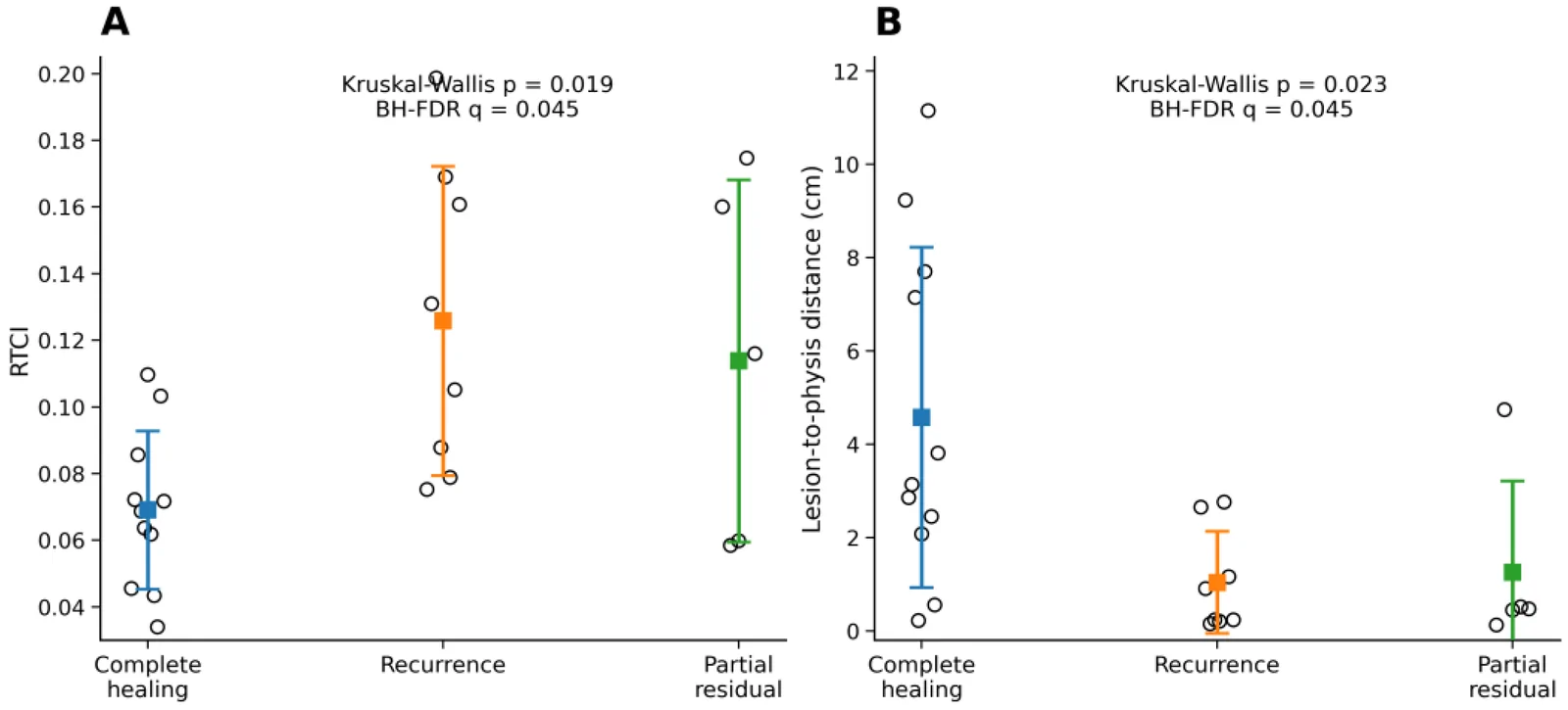
Quantitative imaging measurements across postoperative radiographic outcome groups: (**A**) Relative transverse cyst index (RTCI). (**B**) Lesion-to-physis distance. Open circles represent individual patients; squares and error bars show mean ± SD. Overall *p* values are from Kruskal–Wallis tests, and *q* values are Benjamini–Hochberg FDR-adjusted.

**Figure 3:**
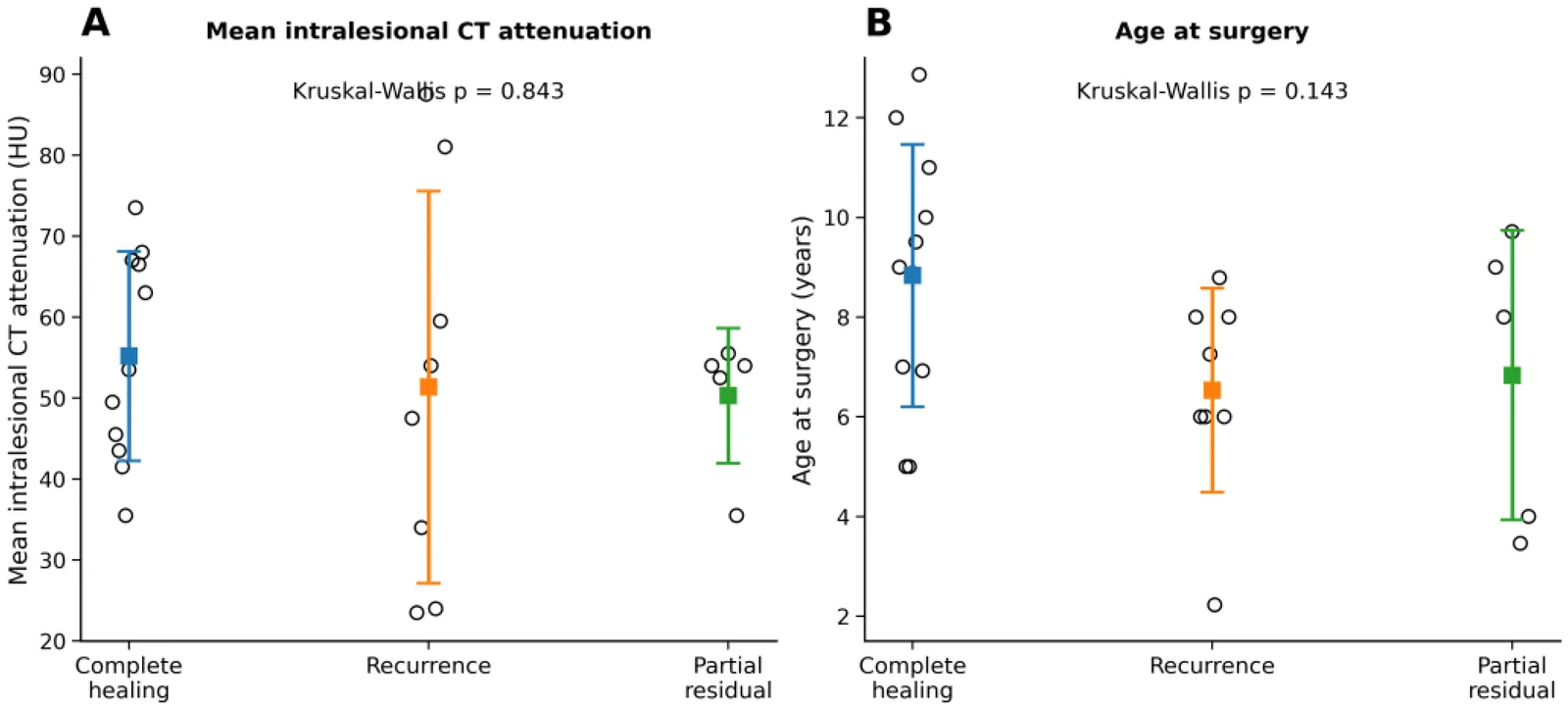
Mean intralesional CT attenuation and age at surgery across postoperative radiographic outcome groups. (**A**) Mean intralesional CT attenuation across the complete-healing, recurrence, and partial-residual groups. (**B**) Age at surgery across the same three outcome groups. Open circles represent individual patients; squares and error bars show mean ± SD. Overall *p* values are from Kruskal–Wallis tests.

**Figure 4:**
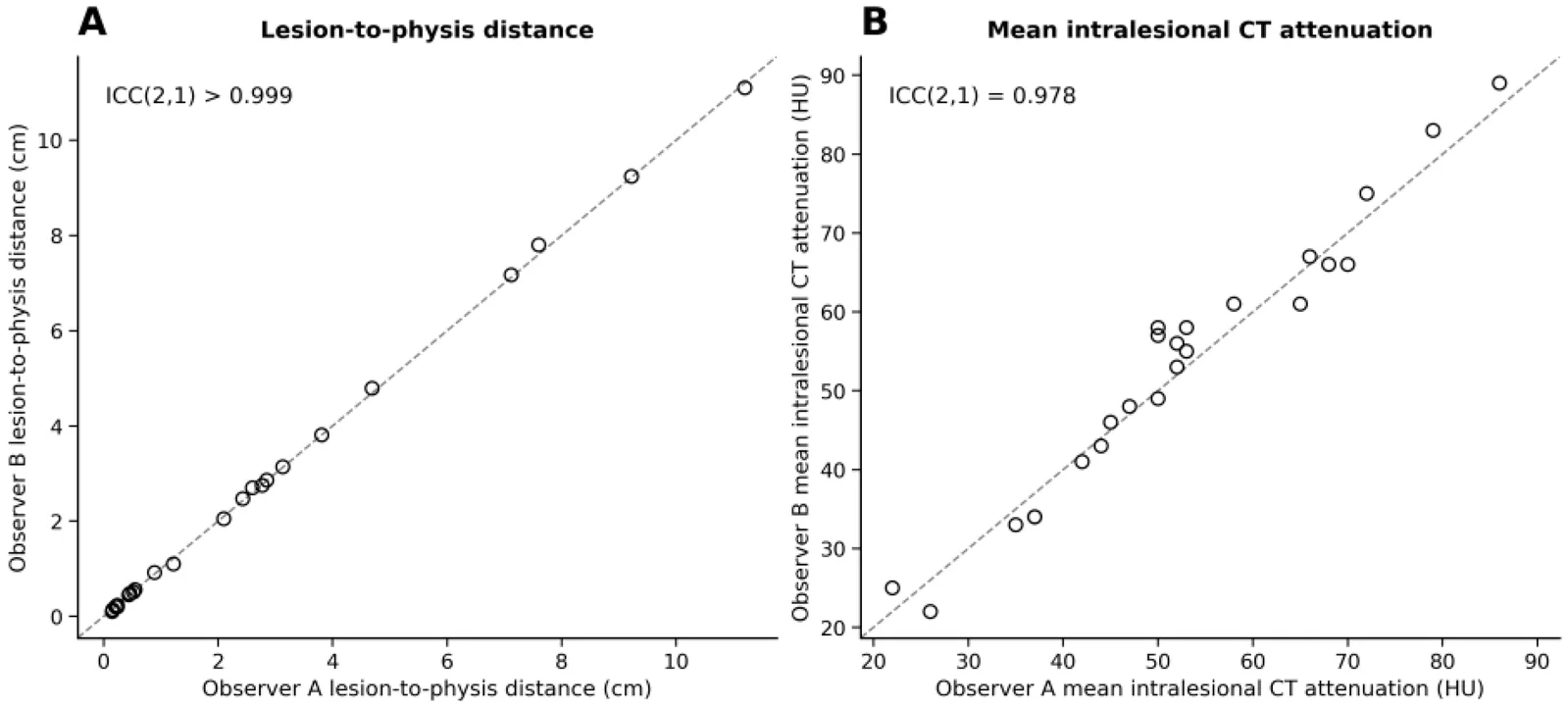
Interobserver agreement for (**A**) lesion-to-physis distance and (**B**) mean intralesional CT attenuation. Each open circle represents one patient; the dashed diagonal line indicates the line of identity. ICC(2,1) was >0.999 for lesion-to-physis distance and 0.978 for CT attenuation.

**Figure 5:**
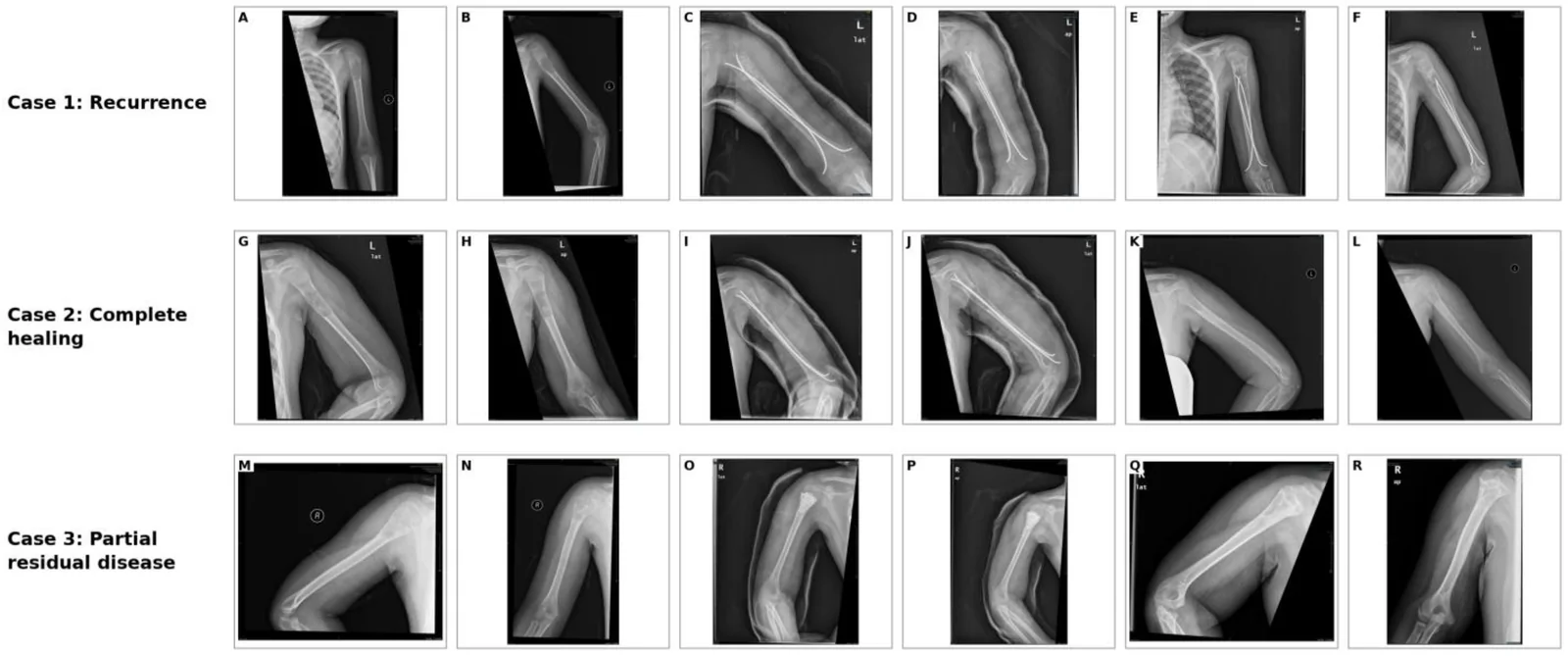
Representative radiographic sequences illustrating the three postoperative radiographic outcome categories: (**A**–**F**) Case 1, recurrence: a 6-year-old boy with a primary aneurysmal bone cyst of the left proximal humerus. Preoperative anteroposterior and lateral radiographs (**A**,**B**) demonstrate an expansile lytic lesion of the proximal humerus. Immediate postoperative radiographs (**C**,**D**) show curettage and bone grafting with elastic intramedullary nail fixation. Follow-up radiographs obtained approximately 37 months after surgery (**E**,**F**) demonstrate recurrent cystic expansion of the proximal humerus, consistent with local recurrence. (**G**–**L**) Case 2, complete healing: an 11-year-old boy with a primary aneurysmal bone cyst of the left humeral shaft. Preoperative radiographs (**G**,**H**) demonstrate an expansile intramedullary lytic lesion of the humeral shaft. Immediate postoperative radiographs (**I**,**J**) show curettage and bone grafting with elastic intramedullary nail fixation. Follow-up radiographs obtained approximately 12.5 months after surgery (**K**,**L**) demonstrate complete osseous consolidation and cortical remodeling without recurrent radiolucency. (**M**–**R**) Case 3, partial residual disease: a 9-year-old boy with a primary aneurysmal bone cyst of the right proximal humerus. Preoperative radiographs (**M**,**N**) demonstrate an expansile metaphyseal lesion of the proximal humerus. Immediate postoperative radiographs (**O**,**P**) show curettage and bone grafting without internal fixation. Follow-up radiographs obtained approximately 24 months after surgery (**Q**,**R**) demonstrate substantial remodeling with a persistent residual radiolucent component but no progressive enlargement, consistent with stable partial residual disease.

**Table 1:** Patient characteristics and treatment outcomes.

Characteristic	*n*	Summary
Age at surgery (years)	24	7.65 ± 2.65
Male sex	19	79.2%
Pathological fracture	16	66.7%
Proximal humerus	9	37.5%
Proximal femur	5	20.8%
Other sites	10	41.7%
Any internal fixation	20	83.3%
Intramedullary nail	15	62.5%
PHP plate	5	20.8%
No internal fixation	4	16.7%
Follow-up (months)	24	36.54 ± 6.88
Complete healing	11	45.8%
Recurrence	8	33.3%
Partial residual disease	5	20.8%

**Table 2:** Prespecified quantitative imaging and clinical variables across postoperative radiographic outcomes.

Variable	Complete Healing (*n* = 11)	Recurrence (*n* = 8)	Partial Residual (*n* = 5)	Overall *p*	BH-FDR *q*
RTCI	0.069 ± 0.024	0.126 ± 0.046	0.114 ± 0.054	0.019	0.045
Lesion-to-physis distance (cm)	4.58 ± 3.64	1.04 ± 1.09	1.26 ± 1.95	0.023	0.045
Mean CT attenuation (HU)	55.2 ± 12.9	51.4 ± 24.2	50.3 ± 8.3	0.843	0.843
Age at surgery (years)	8.83 ± 2.63	6.53 ± 2.05	6.83 ± 2.90	0.143	0.191

Values are mean ± SD. Overall *p* values are from Kruskal–Wallis tests. BH-FDR *q* values correct the four prespecified overall comparisons.

**Table 3:** Interobserver reliability of quantitative imaging measurements.

Measurement	ICC(2,1)	Bootstrap 95% CI
Maximum transverse diameter	0.979	0.953–0.989
Maximum longitudinal diameter	0.999	0.999–1.000
Mean intralesional CT attenuation	0.978	0.947–0.989
Bone length	>0.999	>0.999–1.000
Lesion-to-physis distance	>0.999	0.999–1.000

**Table 4:** Sensitivity analysis comparing complete healing with the composite unfavorable radiographic outcome.

Variable	Complete Healing (*n* = 11)	Unfavorable Outcome (*n* = 13)	Mann–Whitney *p*	BH–FDR *q*
RTCI	0.069 ± 0.024	0.121 ± 0.048	0.0077	0.015
Lesion-to-physis distance (cm)	4.58 ± 3.64	1.12 ± 1.41	0.0065	0.015
Mean intralesional CT attenuation (HU)	55.2 ± 12.9	51.0 ± 19.1	0.582	0.582
Age at surgery (years)	8.83 ± 2.63	6.65 ± 2.30	0.059	0.079

RTCI, relative transverse cyst index; CT, computed tomography; HU, Hounsfield unit; BH–FDR, Benjamini–Hochberg false discovery rate; SD, standard deviation.

## Data Availability

The datasets generated and/or analyzed during the current study are not publicly available because of patient privacy restrictions but may be available from the corresponding authors upon reasonable request and subject to institutional approval.

## Abbreviations

The following abbreviations are used in this manuscript:

ABC	Aneurysmal bone cyst
CT	Computed tomography
MRI	Magnetic resonance imaging
RTCI	Relative transverse cyst index
HU	Hounsfield unit
ROI	Region of interest
ICC	Intraclass correlation coefficient
FDR	False discovery rate
BH-FDR	Benjamini–Hochberg false discovery rate
SD	Standard deviation
